# *Atribacteria* from the Subseafloor Sedimentary Biosphere Disperse to the Hydrosphere through Submarine Mud Volcanoes

**DOI:** 10.3389/fmicb.2017.01135

**Published:** 2017-06-20

**Authors:** Tatsuhiko Hoshino, Tomohiro Toki, Akira Ijiri, Yuki Morono, Hideaki Machiyama, Juichiro Ashi, Kei Okamura, Fumio Inagaki

**Affiliations:** ^1^Geomicrobiology Group, Kochi Institute for Core Sample Research, Japan Agency for Marine-Earth Science TechnologyNankoku, Japan; ^2^Research and Development Center for Submarine Resources, Japan Agency for Marine-Earth Science TechnologyNankoku, Japan; ^3^Faculty of Science, University of the RyukyusNishihara, Japan; ^4^Atmosphere and Ocean Research Institute, The University of TokyoTokyo, Japan; ^5^Department of Marine Resource Science, Faculty of Agriculture and Marine Science, Kochi UniversityNankoku, Japan; ^6^Research and Development Center for Ocean Drilling Science, Japan Agency for Marine-Earth Science TechnologyYokohama, Japan

**Keywords:** submarine mud volcano, deep subseafloor biosphere, methane, *Atribacteria*, digital PCR, CARD-FISH

## Abstract

Submarine mud volcanoes (SMVs) are formed by muddy sediments and breccias extruded to the seafloor from a source in the deep subseafloor and are characterized by the discharge of methane and other hydrocarbon gasses and deep-sourced fluids into the overlying seawater. Although SMVs act as a natural pipeline connecting the Earth’s surface and subsurface biospheres, the dispersal of deep-biosphere microorganisms and their ecological roles remain largely unknown. In this study, we investigated the microbial communities in sediment and overlying seawater at two SMVs located on the Ryukyu Trench off Tanegashima Island, southern Japan. The microbial communities in mud volcano sediments were generally distinct from those in the overlying seawaters and in the well-stratified Pacific margin sediments collected at the Peru Margin, the Juan de Fuca Ridge flank off Oregon, and offshore of Shimokita Peninsula, northeastern Japan. Nevertheless, in-depth analysis of different taxonomic groups at the sub-species level revealed that the taxon affiliated with *Atribacteria*, heterotrophic anaerobic bacteria that typically occur in organic-rich anoxic subseafloor sediments, were commonly found not only in SMV sediments but also in the overlying seawater. We designed a new oligonucleotide probe for detecting *Atribacteria* using the catalyzed reporter deposition-fluorescence *in situ* hybridization (CARD-FISH). CARD-FISH, digital PCR and sequencing analysis of 16S rRNA genes consistently showed that *Atribacteria* are abundant in the methane plumes of the two SMVs (0.58 and 1.5 × 10^4^ cells/mL, respectively) but not in surrounding waters, suggesting that microbial cells in subseafloor sediments are dispersed as “deep-biosphere seeds” into the ocean. These findings may have important implications for the microbial transmigration between the deep subseafloor biosphere and the hydrosphere.

## Introduction

The subseafloor environment is one of the largest biospheres on Earth, with an estimated total abundance of microorganisms of about 2.9 × 10^29^ cells, which is equivalent to half of the microbial cells worldwide in the oceans (Kallmeyer et al., 2012). Over the past decade, subseafloor life and the deep biosphere have been intensively explored by means of scientific ocean drilling, which has assessed the biosphere extent down to around 2.5 km below the ocean floor (Orcutt et al., 2013; Ciobanu et al., 2014; Inagaki et al., 2015). Furthermore, evidence is accumulating that some subseafloor microbial cells are not dead but rather are physiologically active or revivable to some degree, and their long-term metabolic activity may contribute to global biogeochemical cycles (Morono et al., 2011; Røy et al., 2012; Hoehler and Jørgensen, 2013). The taxonomic composition of microbial communities in subseafloor sedimentary habitats has been extensively studied using a variety of cultivation-dependent and -independent molecular approaches, demonstrating that some predominant microbial taxa, distinct from known water-column communities, widely inhabit sediments at a variety of oceanic locations under similar geochemical and geophysical settings (e.g., Inagaki et al., 2003, 2006; Webster et al., 2004; Biddle et al., 2008). One of the common predominant microbial taxa frequently found in the marine sediment is *Atribacteria* including members of JS1 and OP9 which are recently revealed to be monophyletic by phylogenomic analyses of single-cell amplified genome (Nobu et al., 2016). *Atribacteria* are especially predominant in anaerobic, organic carbon replete, and methane-rich marine sediment. Although representatives of this phylum have not been cultivated as yet, several recent studies of single-cell amplified genomes consistently suggested anaerobic heterotrophic metabolism (Carr et al., 2015; Nobu et al., 2016). Although the co-occurrence of *Atribacteira* with methane (Inagaki et al., 2006), no evidence has been reported that this phylum was directly involved in methane production or consumption. Alternatively, *Atribacteira* would control methane production in anaerobic marine sediment by primary or secondary fermentation producing fermentation products as acetate or CO_2_, that can be used as substrates for methanogen (Carr et al., 2015; Nobu et al., 2016).

Among diverse geological and oceanographic settings in the ocean, numerous submarine mud volcanoes (SMVs) have been observed worldwide along the margins of convergent plates, which transport mud, gasses and fluids from several kilometers below the seafloor to the overlying hydrosphere (Milkov, 2000; Kopf, 2002 and references therein). SMVs accordingly serve as conduits for the release of substantial amounts of methane and other hydrocarbons into the water column (Sauter et al., 2006; Wallmann et al., 2006; Sahling et al., 2009), and hence act as natural pipelines in the geosphere. Over the past decades, the microbial communities inhabiting hydrocarbon seeps associated with SMVs have been investigated around the world, including at the Håkon Mosby (Niemann et al., 2006; Jerosch et al., 2007; Losekann et al., 2007), Kazan (Kormas et al., 2008; Pachiadaki et al., 2010), Amsterdam (Pachiadaki et al., 2011), Chefren (Omoregie et al., 2008), and Gulf of Mexico mud volcanoes (Martinez et al., 2006) and at the Nankai Trough (Miyazaki et al., 2009). The studies at SMVs mainly focused on the anaerobic oxidation of methane (AOM) consortia of sulfate-reducing bacteria and methane-oxidizing archaea living in sediments at the sulfate–methane transition zone (SMTZ). Considering that SMVs transport not only solid materials (e.g., muds and breccias) but also gasses and fluids from deep sources (Sauter et al., 2006; Feseker et al., 2008), it is possible that submarine mud volcanism may also transport microbial communities from the deep subseafloor biosphere to the overlying ocean.

Deep-sea hydrothermal vents on the ridge systems have been proposed as “windows to a subsurface biosphere” (Deming and Baross, 1993), and there have been some investigations into the dispersal of subseafloor microbial communities, including mesophilic to hyperthermophilic microbes, through volcanic and hydrothermal vent activity (e.g., Huber et al., 2003, 2006; Takai et al., 2004; Jungbluth et al., 2016). In the hydrothermal systems, however, only surface of vents are habitable zone for microbes due to the very steep temperature gradient up to higher than 300°C. Therefore, deep-sea hydrothermal vents would not be considered as conduits of deep biosphare and hence the geosphere–biosphere interactions at sedimentary geosystems (e.g., oceanic plate subduction zones, forearc sedimentary basins) as well as interactions between the Earth’s surface and deep subseafloor biospheres in the large oceanic province remain largely unknown.

In the present study, we investigated microbial communities inhabiting two SMVs off Tanegashima Island, Japan (Ujiie, 2000), by taking sediment cores from the summit (down to a sediment depth of 3.6 m) and samples of overlying seawater (up to 200–300 m above the summit). Sediment core samples were collected in 2015 using a Navigable Sampling System (see Ashi et al., 2014), which enables pinpoint piston coring with television-monitoring of the seafloor. The concentrations of methane in the water column peaked at 19 and 72 m above the two SMVs, respectively, indicating the presence of a methane plume from the SMV. To identify and quantify members of the microbial communities in mud volcano sediment and seawater samples, we applied molecular analysis of 16S rRNA genes using a second generation sequencer, image-based cell counts, catalyzed reporter deposition-fluorescence *in situ* hybridization (CARD-FISH), and microfluidic digital PCR techniques.

## Materials and Methods

### Geological Settings

**FIGURE 1:**
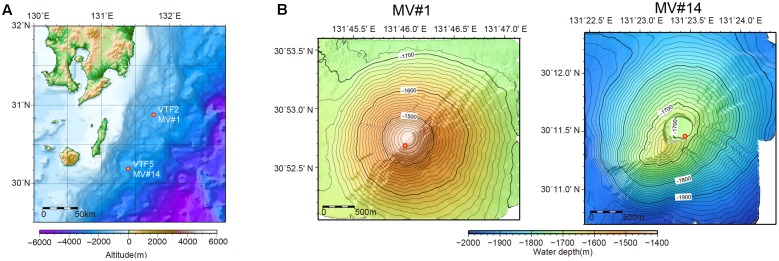
**(A)** Location of submarine mud volcanoes (SMVs) MV#1 and MV#14 along the Ryukyu Trench near Tanegashima Island, Japan. **(B)** Detailed bathymetric maps of MV#1 and MV#14. Both SMVs are about 3000 m in diameter and 300 m in height. The yellow circles indicate sampling points.

Previous acoustic seafloor surveys identified several 10s of SMVs in the northwestern Pacific Ocean at water depths of 1200–3500 m off Tanegashima Island, Japan. For one of these, geochemical information, including stable isotopic compositions of methane and water (i.e., interstitial water in sediment) is available (Nakayama et al., 2010), whereas there are as yet no microbiological studies. In 2015, we investigated the distribution and diversity of microbial communities inhabiting two of the SMVs in this area: MV#1 and MV#14 (**Figure 1**). MV#1 has a diameter of about 3000 m, a height of 300 m, and is situated at a water depth of 1419 m. MV#14 has a diameter of about 3000 m, a height of 250 m, and is situated at a water depth of 1691 m. Intensive seafloor surveys around MV#1 using the autonomous underwater vehicle *Urashima* and remotely operated vehicle *Hyper-Dolphin* (Japan Agency for Marine-Earth Science Technology [JAMSTEC]) showed well-preserved mud-flow channels suggestive of a recent eruption.

### Sampling of Mud Volcano Sediment and Seawater

Sediment and seawater samples were obtained at two stations, VTF2 (MV#1) and VTF5 (MV#14), during cruise KH-15-2 of R/V *Hakuho Maru* in 2015. Sediment samples were collected using the Navigable Sampling System (Atmosphere and Ocean Research Institute, The University of Tokyo). At each station, hydrographic parameters were recorded with a CTD-carousel system (SBE 9, Sea-Bird Scientific, Bellevue, WA, United States), which was deployed with simultaneous water sampling using Niskin bottles (General Oceanics, Inc., Miami, FL, United States). For comparison in the DNA analysis, we used stratified sediment core samples from the Peru Margin, Juan de Fuca ridge flank, and offshore of the Shimokita Peninsula, Japan, which were collected during Ocean Drilling Program (ODP) Leg 201 in 2002, Integrated Ocean Drilling Program (IODP) Expedition 301 in 2004, and shakedown cruise CK06-06 of JAMSTEC’s deep-sea drilling vessel (D/V) *Chikyu* in 2006, respectively (**Table 1**). For DNA extraction, all SMV sediments and the stratified sediments were frozen immediately after sampling and stored at -80°C until use. For cell count of the SMVs sediment, 2 g of sediment was collected by using a tip-cut syringe and transferred to a 15-ml falcon tube. Subsequently, 8 ml of 4% paraformaldehyde was added to the sediment for fixation. After overnight fixation at 4°C, the fixed sediment was washed twice with PBS buffer and stored in 50% PBS/ethanol at -20°C.

**Table 1:** Sampling sites.

Cruise/expedition	Site	Latitude	Longitude	WD^∗^	SD^∗∗^
KH-15-02	VTF2 (MV#1)	30.88	131.77	1401	18–356
	VTF5 (MV#14)	30.19	131.39	1667	29–301
ODP Leg 201	1227	-8.99	-79.96	427	50–660
IODP Expedition 301	1301C	47.75	-127.76	2656	20–370
CK-06-06	C9001	41.18	142.20	1180	100–280


*^∗^WD, water depth [m]; ^∗∗^SD, depth of the sampled sediment [cm below seafloor].*

### Methane and Sulfate Concentrations in Sediment Pore Water

After core recovery, wet sediment was immediately sampled at 20- to 50-cm intervals and pore water samples were extracted onboard with a stainless-steel squeezer (Manheim and Sayles, 1974). The sulfate content of pore water was analyzed using an ion chromatograph (Dionex ICS-500) with an isocratic carbonate/bicarbonate eluent coupled with suppressed conductivity detection. Analytical precision was estimated to be within 0.4% from repeated measurements of the same samples.

Sediment samples for the determination of methane concentrations were collected using tip-cut plastic syringes immediately after core recovery. The syringe end was tightly capped with a silicone-rubber stopper. The sediment sample was transferred from the syringe into a glass vial in a N_2_-flushed glove bag, and then the vial was capped with a Teflon-coated rubber septum and aluminum seal for subsequent geochemical analyses. The glass vials were frozen at -20°C and stored until analysis. In a laboratory on shore, the glass vials were heated in an oven at 60°C for 30 min. From the glass vials, 0.1–0.5 mL of the gas phase was extracted with a gas-tight syringe and introduced into a gas chromatograph-isotope ratio monitoring mass spectrometer (Tsunogai et al., 2002). The methane content was calculated by comparing the ^44^CO_2_ output of the isotope-ratio-monitoring mass spectrometer with those measured during analyses of a working-standard gas containing 5000 ppm methane in He. The standard deviation of repeated analysis of the laboratory standard gas was <4%.

### Methane Concentration in Seawater

For quantification of methane in seawater, two replicate samples were collected from each Niskin bottle: two 120-ml serum vials were filled to overflowing after flushing with approximately two vial-volumes to avoid air contamination. The seawater samples were fixed by the addition of 500 μl saturated HgCl_2_ solution to inhibit microbial activity during storage, sealed with gray butyl-rubber septa and aluminum crimp-caps, and then stored in the dark at 4°C until analysis in the laboratory at the University of the Ryukyus (Okinawa, Japan).

Dissolved methane was analyzed by purge–trap gas chromatography (GC) (GC-2014, Shimadzu) equipped with a flame ionization detector. Hydrocarbons were chromatographically separated on a Porapak Q packed column (80–100 mesh; 2-m length) operated at 40°C using ultra-high-purity He as the carrier gas (Air Liquide, purity 99.9999%). The system was calibrated using a gas standard with a certified methane concentration (9.72 ppm) provided by Japan Fine Products (Kawasaki, Japan). The precision of the method was 10%, as estimated from the standard deviation of replicate analysis of water samples (*n* = 4).

### Cell Counts

Cell concentrations in sediment samples were determined using a previously described procedure (Morono et al., 2013). Briefly, after washing fixed sediment with a NaCl solution, a mixture of detergent and methanol was added and the sediment slurry was shaken for 60 min at 500 rpm using a Shake Master (Bio Medical Science, Tokyo, Japan). The slurry was then sonicated and carefully layered onto a high-density cushion consisting of multiple density layers of Nycodenz and sodium polytungstate solutions. Following centrifugation at 10,000 × *g* for 60 min, the supernatant was carefully transferred to a new vial. The detergent and methanol mixture was added to the remaining pellet, and the mixture was again sonicated and centrifuged with the high-density cushion solution. The supernatants were pooled and the combined recovered suspension was filtered onto a polycarbonate membrane. For seawater samples, 300 mL of formalin-fixed seawater was directly filtered onto a polycarbonate membrane. The number of microbial cells stained by SYBR Green I was determined either using an automated cell counting system with analysis of acquired images using MetaMorph software (Molecular Devices, Sunnyvale, CA, United States) (Morono and Inagaki, 2010), or counted manually under fluorescence microscopy.

### DNA Extraction

DNA was extracted from 5 g wet sediment or 300 mL seawater using the PowerMax Soil DNA Isolation Kit (Qiagen, Tokyo, Japan) according to manufacturer’s instructions. For the seawater samples, soon after recovery of the Niskin bottles 300 mL seawater was filtered using a disposable MicroFunnel 300 Filter Unit with 0.2-μm pore size (Pall Corporation, Tokyo, Japan) and stored at -20°C prior to extraction. For a negative control, 5 mL Milli-Q water (Millipore) was used for extraction in place of sediment or seawater. The extracted DNA solution (approximately 5 mL) from sediment was further concentrated by ethanol precipitation. To increase the DNA yield, 10 μL of linear acrylamide (Thermo Fisher Scientific, Kanagawa, Japan) was added before centrifugation. The DNA precipitate was dissolved in TE buffer (10 mM Tris-HCl, 1.0 mM EDTA; pH 8.0) and stored at -20°C until use.

### Quantification of 16S rRNA Genes

The abundance 16S rRNA genes was quantified by microfluidic digital PCR (dPCR) using the BioMark Real-time System and 12.765 Digital Array (Fluidigm Corporation, South San Francisco, CA, United States) as described elsewhere (Hoshino and Inagaki, 2012). For each sample, 6 μL of PCR mixture was prepared as follows: 3.0 μL of 2× MightyAmp buffer (TaKaRa Bio, Shiga, Japan), 0.15 μL of Binding Dye sample loading reagent (Fluidigm), 0.12 μL of MightyAmp DNA polymerase (TaKaRa Bio), 0.3 μL of EvaGreen (Biotum, Fremont, CA, United States), 0.015 μL of Rox dye, 0.15 μL each of forward and reverse primer, 1.0 μL of extracted DNA, and 1.115 μL of water. We used the domain-specific primers B27F-B357R and A806F-A958R (**Table 2**) for the amplification of bacterial and archaeal 16S rRNA genes, respectively.

**Table 2:** Oligonucleotide primers and *Atribacteria*-specific probe used in this study.

Primer	Sequence (5′–3′)	Target	Use^∗^	Reference
U515F	TGYCAGCMGCCGCCGTAA	Prokaryote	S	Hoshino and Inagaki, 2017
U806R	GGACTACHVGGGTWTCTAAT	Prokaryote	S	Walters et al., 2011
515F_miseq^∗∗^	AATGATACGGCGACCACCGAGATCTACACTAGATCGCTCGTCGGCAGCG	1 st PCR product	S	Caporaso et al., 2012
	TCAGATGTGTATAAGAGACAGRYSWRTGYCAGCMGCCGCGGTAA			
806R_miseq	CAAGCAGAAGACGGCATACGAGAT[index12nt]GTCTCGTGGGCTCGGAGAT	1 st PCR product	S	Caporaso et al., 2012
	GTGTATAAGAGACAGRYSWRGGACTACHVGGGTWTCTAAT			
B27F	AGRGTTYGATYMTGGCTCAG	Bacteria	D	Lane, 1991
B357R	CTGCWGCCNCCCGTAGG	Bacteria	D	Herlemann et al., 2011
A806F	ATTAGATACCCSBGTAGTCC	Archaea	D	Raskin et al., 1994
A958R	YCCGGCGTTGAMTCCAATT	Archaea	D	DeLong, 1992
Atri578	ACTTTTAAGACCGCCTACGA	*Atribacteria*	C	This study


*^∗^S, sequencing; D, digital PCR; C, CARD-FISH. ^∗∗^Index sequence is underlined.*

### Sequencing and Data Processing

The V3–V4 hyper-variable region of the 16S rRNA gene, which is often used for analyzing microbial diversity and community structure, was amplified by PCR using universal primers U515F/U806R (Table 2). The 25-μl PCR mixture consists of 0.3 μM of each primer, 1 μl of the template DNA, 1× MightyAmp Buffer Ver.2, and 0.5 μl of MightyAmp DNA polymerase (TaKaRa Bio). The PCR started with 2 min at 98°C followed by 30 cycles of denaturation at 98°C for 10 s, annealing at 55°C for 15 s, and elongation at 68°C for 30 s. After purification of the desired PCR products by agarose gel electrophoresis, index and adapter were added to the purified product during the eight cycles of second-round PCR using KAPA HiFi HotStart Ready mix (Kapa Biosystems) with 250 pg of the purified PCR product as described elsewhere (Caporaso et al., 2012). The indexed PCR products were purified twice by AMPure XP (Beckman Coulter), and then the PCR product was sequenced by the MiSeq platform with MiSeq Reagent Kit v3, 600 cycles (Illumina, San Diego, CA, United States). All the purified PCR products were sequenced in a sequencing run of Miseq and account for two fifth of total clusters. Raw sequences were demultiplexed, and the sequence quality was filtered using the BaseSpace app (Illumina). Further processing, including primer trimming, sequence length screening, taxonomic identification, clustering and diversity analysis, were performed using the ‘mothur’ software package (v. 1.35.0) (Schloss et al., 2009) and USEARCH 64-bit version^1^. After the screening, the average number of sequence reads per sample was 54,410 (Supplementary Table 2). The operational taxonomic units (OTUs) obtained were used for non-metric multidimensional scaling (nMDS) analysis using the ‘vegan’ package of R software as described elsewhere (Dixon, 2003; Doi and Okamura, 2011) for beta-diversity analysis of microbial communities. To exclude confounding results due to potential contaminants from the extraction process and chemicals used for PCR and sequencing, the top 10 families in the negative controls, and previously reported core-human microbiomes (Li et al., 2013) were arbitrary removed (listed in Supplementary Table 1). The removed top 10 families are generally consistent with laboratory and reagent contaminants in microbiome analyses (Salter et al., 2014). We performed all the analyses in this study without removing those potential contaminant and confirmed that removal of those did not significantly affect the results (Supplementary Figures 1, 2 and Table 2).

### CARD-FISH for *Atribacteria*

To detect and visualize atribacterial cells in microbial communities in the MVs, we designed a new oligonucleotide probe Atri578 (**Table 2**), which can be used to detect a broad range of atribacterial sequences in the ARB Silva Database by using ARB software and mathFISH (Ludwig et al., 2004; Yilmaz et al., 2011). The coverage of Atri578 to atribacterial sequences determined by TestProbe3.0 with the SILVA Database SSU128 is 70.6% and only two sequences in the outgroup are no-mismtach to Atri578. The horseradish peroxidase (HRP)-labeled Atri578 probe was purchased from Biomers GmbH (Ulm, Germany). The optimal formamide concentration in hybridization buffer was determined by clone-FISH (Schramm et al., 2002) using a synthesized vector (Eurofins Genomics, Tokyo, Japan) containing the full-length 16S rRNA gene sequence of *Atribacteria* bacterium SCGC AAA255-N14 (ASPC01000002). We tested formamide concentrations of 0% to 60% at 10% intervals, at 35°C and found 10% to be the optimal formamide concentration. Using this concentration, *Atribacteria* on the membrane filters were detected by CARD-FISH for the water column samples. The sediment samples from SMVs had very low cell number and the number of cells on the filter membrane after cell separation described above was not enough to detect *Atribacteria*.

Catalyzed reporter deposition-fluorescence *in situ* hybridization was performed according to a previously described protocol (Hoshino et al., 2008, 2015). Briefly, hybridization was performed in hybridization buffer containing 10% formamide and 0.5 μM of the HRP-labeled Atri578 probe at 35°C for 2 h. After washing away excess probe, the membrane filter was incubated with Alexa555- or Alexa488-labeled tyramide (Thermo Fisher Scientific) at 35°C for 30 min for signal deposition, followed by counterstaining with SYBR Green I or DAPI.

### DNA Accession Numbers

Sequence data obtained in this study have been submitted to the DDBJ database under accession numbers DRA005491 and DRA005492.

## Results

### Methane and Sulfate Concentrations

**FIGURE 2:**
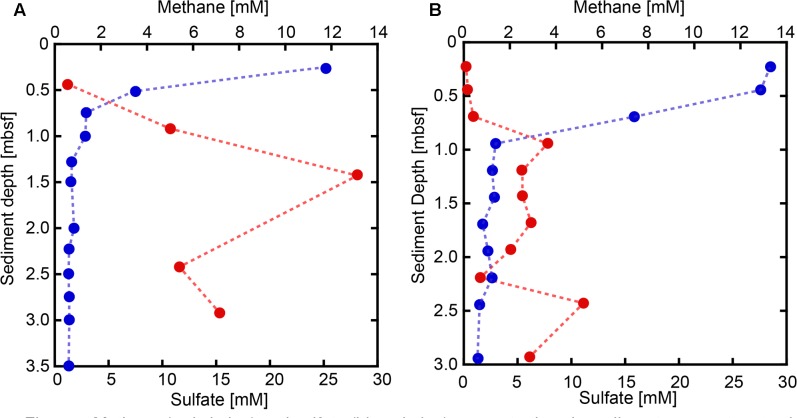
Methane (red circles) and sulfate (blue circles) concentrations in sediment pore water at SMVs MV#1 **(A)** and MV#14 **(B)**.

Sulfate concentrations in sediment pore water from both SMVs showed maxima around 25–30 mM in the uppermost sediment, with concentrations rapidly decreasing with depth (**Figure 2**). In sediments deeper than 1 m below the seafloor (mbsf), sulfate was almost depleted at both SMVs. The concentration of methane was generally higher in pore water from MV#1 than MV#14, ranging from 0.5 to 13 mM and from 0.06 to 5.1 mM, respectively. Although measured concentrations varied widely probably because of depressurization of sediment, methane was depleted near the sediment surface at around 1 mbsf. The depth profiles of sulfate and methane indicate that the SMTZ was located at around 1 mbsf at both SMVs. In the overlying water column, the concentration of methane peaked to 0.71 nM at 29 m above MV#1 and to 1.93 nM at 70 m above MV#14. Those peaks indicate the occurrence of methane plume and thus advective flow from the SMVs because methane is immediately oxidized in water column and its concentration almost never reached to that high in normal water column at the similar depth (Toki et al., 2016).

### Cell Counts and 16S rRNA Gene Abundance

**FIGURE 3:**
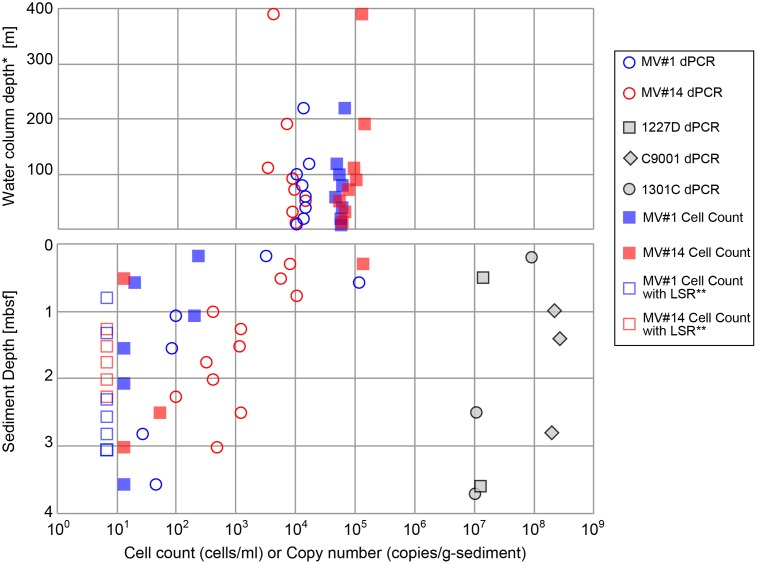
Microbial cell counts by SYBR Green I staining, and digital PCR (dPCR) quantification of 16S rRNA gene abundance. dPCR results are the sum of bacterial and archaeal 16S rRNA gene abundance. dPCR results from reference sites from previous drilling expeditions are provided for comparison (see **Table 1** for sampling site information). ^∗^Height above the summit of the SMVs. ^∗∗^LSR, “low statistical reliability” because of very low, but detectable, cell numbers.

Cell densities in seawater, based on counts of cells stained with SYBR Green I, were estimated to be in the range of 4.7 × 10^4^ to 1.4 × 10^5^ cells/ml at MV#1 and MV#14, respectively (**Figure 3**). The gene abundance of 16S rRNA genes in seawater, as sum of archaeal and bacterial 16S rRNA gene (Supplementary Figure 3) quantified by microfluidic dPCR, were overall slightly lower than the cell densities. Cell counts and gene abundance from the seawater above the SMVs were relatively constant throughout the water column at 5 × 10^3^ copies/ml and 6 × 10^4^ cells/ml (Top panel of **Figure 3**) up to around 400 m above the summit, the upper limit of sample collection.

The cell concentrations in the shallowest sediment samples from the seafloor were 2.4 × 10^2^ and 1.3 × 10^5^ cells/g-wet sediment for MV#1 and MV#14, respectively. Cell concentrations at the two SMVs dropped sharply to only 20 and 13 cells/g-wet sediment at 0.58 and 0.51 mbsf, respectively. The 16S rRNA gene abundance for MV#1 in the uppermost sediment sample was 3.3 × 10^3^ copies/g-wet sediment, which is an order of magnitude higher than the cell concentration, whereas the cell counts in the uppermost sediment at MV#14 were higher than the 16S rRNA gene abundance (8.0 × 10^3^ copies/g-wet sediment). The 16S rRNA gene abundance in sediment from depths greater than 1.5 mbsf were 10–100 times the cell concentrations.

Throughout the sediment cores, MV#1 generally had lower 16S rRNA gene abundance than MV#14, and the gene abundance at MV#1 decreased to about 1 × 10^2^ copies at 1.1 mbsf. At MV#14, the 16S rRNA gene abundance gradually decreased with increasing depth to 4.7 × 10^2^ copies/g-wet sediment at 3.0 mbsf, the deepest sediment sampled. The estimated 16S rRNA gene abundance as well as cell concentrations in the deeper mud volcano sediments were markedly lower than those in shallow seafloor sediments and in other stratified subseafloor sediments on Pacific margins collected during previous IODP expeditions. In general, the abundance of 16S rRNA genes in the SMVs varied from 10^1^ to 10^5^ copies/g-wet sediment, which is two to seven orders of magnitude lower than in stratified sedimentary environments (**Figure 3**).

The cell counts are higher than the gene abundance in the water column in this study, whereas in the sediment the trend was opposite to the water column. The reasons of formar discrepancy can be partially explained by biases as low DNA extraction efficiency or false positive counts of fluorescence-emitting particles (Lloyd et al., 2013). The latter might be caused by low recovery of cells from very sticky texture of the MV sediment. In any case, the results from both methods are consistent with the general trends; (1) Higher microbial abundance in the overlying seawater than mud volcano sediment: (2) Decrease of microbial abundance with sediment depth.

### Microbial Community Composition of Mud Volcano Sediments

**FIGURE 4:**
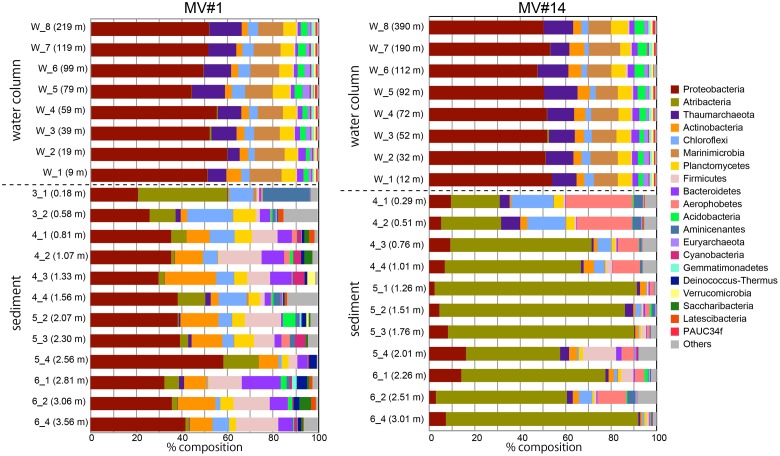
Microbial community composition in the sediment and overlying seawater at two SMVs using phylum-level classification. Only the top 20 phyla are shown. Numbers in parentheses show the water column depth (meters above the SMV summit) for water samples (labels that start with “w”) and depth in sediment for sediment samples (labels that start with numbers).

We obtained around 1.6 million sequences in total by Miseq sequencing of SMV sediment and overlying seawater samples (Supplementary Table 2). Phylum-level classification showed the predominance of *Proteobacteria* in all seawater samples, comprising more than 50% in all sequence libraries (**Figure 4**). The taxonomic composition of microbial communities in seawater was generally constant at both sites and at different depths. The second most representative phylum was *Marinimicrobia* (formerly SAR405), comprising 10–14% of the total community, followed by *Thaumarchaeota* comprising 5–15%.

In contrast, the community composition of mud volcano sediments was markedly different from that in the water column, even at the phylum level; for example, *Marinimicrobia* was nearly absent in sediment samples. The MV community composition was also distinct from those of the reference stratified sediment (Supplementary Figure 4). In addition, the community compositions at the two mud volcano sites were notably different: *Proteobacteria* was dominant at MV#1, comprising between 21 and 58% of the total, whereas *Atribacteria* (formerly OP9 and JS1) comprised 21–88 and 0.7–39% of total sequence reads at MV#14 and MV#1, respectively. Some phyla, such as *Firmicutes, Aerophobetes* (formerly CD12), and *Aminicenantes* were prevalent only in the sediment samples.

### Beta-Diversity of Microbial Communities in Sediment and Seawater Samples

**FIGURE 5:**
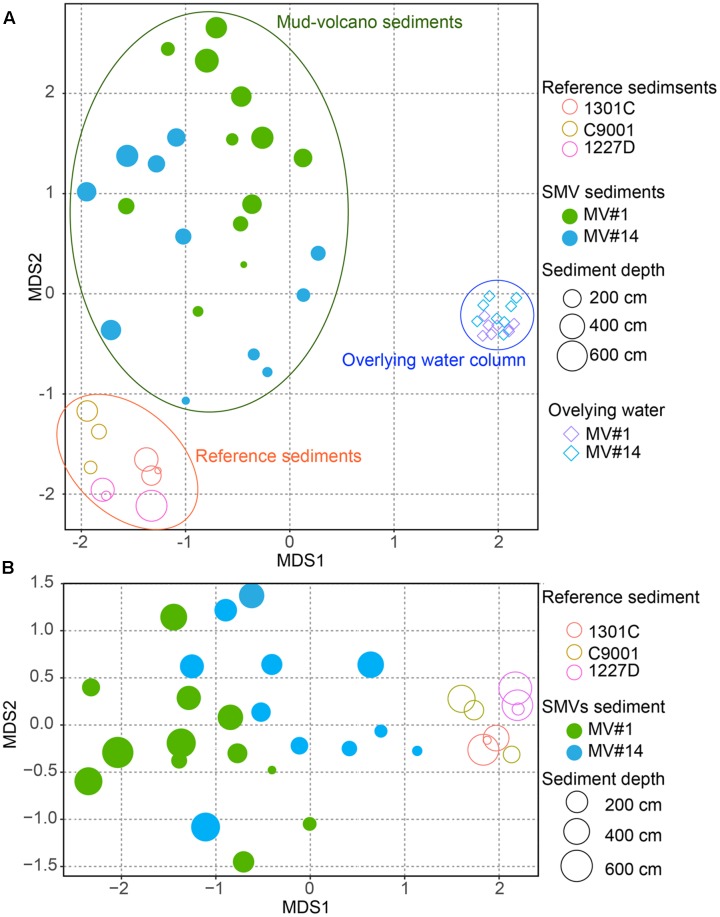
Non-metric multidimensional scaling (NMDS) plot of microbial communities from SMV sediment (circles) and the overlying water column (diamonds), with stratified marine sediment communities included for reference, based on operational taxonomic unit (OTU) composition. The size of the circles for sediment samples, including SMV sediment is proportional to sediment depth. **(A)** NMDS plot of all the samples including the water column. **(B)** Only SMV and reference sediments.

The degree of similarity between microbial communities in sediment and seawater was determined by nMDS analysis applied to OTUs with >97% similarity (**Figure 5**). Microbial communities in the overlying seawater from 200 to 300 m above the mud volcano summits were generally similar. Compared to the seawater communities, microbial communities in sediments were more diverse, even across very small changes in depth at 2 and 6 mbsf for the SMVs and reference stratified marginal sediments. Microbial communities in stratified sediments were grouped into one cluster despite being collected from geographically distinct sites, whereas the microbial communities in SMV sediments were different from those in both the stratified sediments and in the overlying water column. A gradation in microbial composition is evident from deep to shallow sediment within both SMVs. These trends suggest that physical and chemical conditions may affect microbial composition more than can be explained by the conditions in well-stratified sediment. The nMDS plots (**Figure 5**) also indicate that shallower SMV sediment communities, especially at MV#14, are similar to those in normal stratified marine sediments. This difference might suggest differences in activity of the SMVs: if MV#1 is more active than MV#14, the seafloor at MV#14 would be covered with marine sediments freshly deposited since the last eruption; consequently, shallower sediments at MV#14 would harbor the microbial communities that typically occur in the surrounding stratified sediments.

### Common Microbial Components of Mud Volcano Sediments and the Overlying Water Column

**FIGURE 6:**
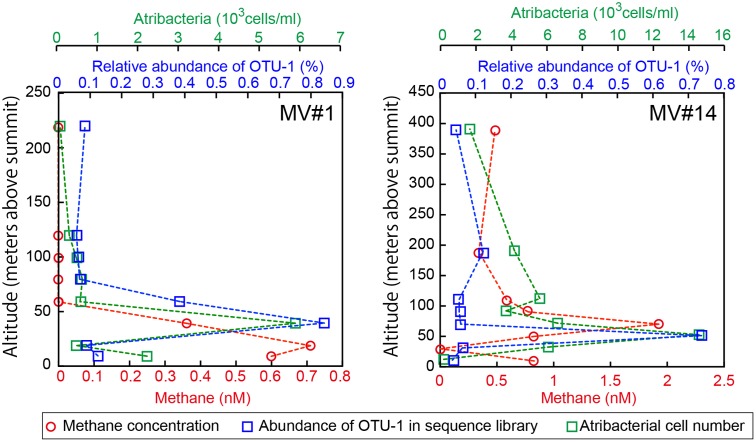
Depth profiles of methane concentrations, relative abundance of OTU-1 in the sequence library, and cell numbers of *Atribacteria* as detected by CARD-FISH using probe Atri578. OTU-1 is the most abundant OTU affiliated with *Atribacteria.*

We identified the common OTUs in all SMV sediment and seawater samples to determine whether mud volcano activity dispersed some of the community members of the subseafloor sedimentary habitats at SMVs to the overlying seawater. We found only one OTU (i.e., OTU-1) common to all of the samples examined for both sites (MV#1 and MV#14). OTU-1 is the most abundant OTU in the SMV sediment samples, comprising 1.4–38 and 22–85% of the sequence reads from MV#1 and MV#14, respectively. OTU-1 sequences are affiliated with *Atribacteria*, which is well-known as a predominant taxonomic clade of marine sedimentary bacteria (e.g., Inagaki et al., 2006; Webster et al., 2006b; Blazejak and Schippers, 2010); almost all *Atribacteria*-related sequences detected in this study are included in this OTU (Supplementary Figure 5). The relative abundance of OTU-1-related sequences was generally low in seawater samples, comprising less than 0.1% of total reads. This abundance peaked, however, to more than 0.7% at 39 m (MV#1) and 52 m (MV#14) above the summits of both SMVs and returned to around 0.1% above this depth (**Figure 6**). Interestingly, the depth profile of methane similarly peaked at 19 m (MV#1) and 72 m (MV#14) above the summit, indicating the presence of *Atribacteria* in methane plumes emanating from the SMVs.

**FIGURE 7:**
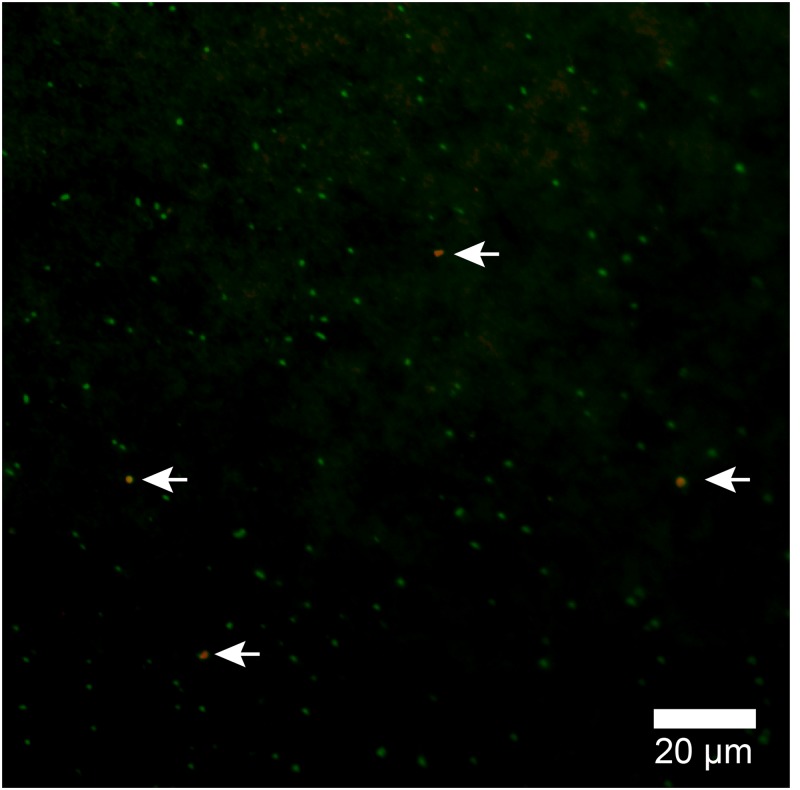
Atribacterial cells detected by CARD-FISH using probe Atri578. Alexa555-labeled tyramide was used for horseradish peroxidase (HRP) detection (appears orange) and DNA was counterstained with SYBR Green I. White arrows point to orange cells (*Atribacteria*); green cells are other microbial cells.

Because the relative abundance of OTU-1 in sequence libraries are not necessarily quantitative, we verified the presence and abundance of *Atribacteria* in seawater samples by CARD-FISH using the newly designed probe Atri578, which specifically targets the *Atribacteria*-related sequences detected in this study and related sequences in publicly available databases (**Figure 7**). CARD-FISH analysis showed that *Atribacteria* in the water column were most abundant at 39 m (MV#1) and 52 m (MV#14) above the respective summits; results overall in good agreement with the relative abundance of OTU-1 sequence. Consequently, we infer that *Atribacteria* inhabiting the subseafloor sediments at SMVs are dispersed into the overlying seawater along with the methane plume.

## Discussion

The mechanisms of dispersal of microbial life from the deep biosphere to the Earth’s surface biosphere remains largely unknown. In this study, we observed that deep-biosphere microbial communities are transported from deep subseafloor sedimentary environments to the ocean through the activity of SMVs; specifically, members of the *Atribacteria*, typically observed in the global subseafloor sedimentary environment, were found to be present in methane plumes above two SMVs. The deep-sea hydrothermal systems have been well-studied and dispersal of thermophilic microbial communities to the ocean has been well-known (Deming and Baross, 1993; Takai et al., 2004; Huber et al., 2006). However, the diffused microbial communities inhabit around the surface of vents due to the high temperature of hydrothemal fluid and those do not come from deep biosphere. In contrast to those systems, little is known about the dispersal and transport of microorganisms from the deep subseafloor sedimentary biosphere to the overlying hydrosphere. A recent molecular ecological study of microbial communities in marine sediments and the overlying seawater showed that some of the OTUs represented by abundant sequences in the sediment are also present but represented by rare sequences in the water column population (Walsh et al., 2016); however, the possible mechanism or the driving force for the dispersal remains unknown. In this study, we examined these scientific questions at two SMVs off Tanegashima Island, Japan.

We found that *Atribacteria*-related sequences were prevalent throughout the sediment and overlying water column at both SMVs examined. One of the atribacterial phylotypes (i.e., OTU-1) was commonly present in all samples. It is possible that only a very small portion of the sedimentary microbial cells can be diffused from sticky mud volcano sediments into the overlying water column, so we might only be able to detect the most abundant deep biosphere-derived OTUs because of the sensitivity of sequencing analysis. In support of this idea, the second most abundant OTU in the sequence libraries of the sedimentary microbial community (OTU-12, within the *Aerophobetes*) was found in the water column and showed a profile similar to those of OTU-1 and *Atribacteria* cell counts (Supplementary Figure 6). These consistent results suggest that there might be no selection of microbial phylotypes in the dispersal process, and hence sedimentary microbes are evenly dispersed to the overlying seawater along with discharging gasses and fluids.

The candidate division JS-1 affiliated with *Atribacteria* was originally found in the sediment from Japan Sea (Rochelle et al., 1994), and meanwhile the widespread distribution of this group was reported in marine sediments including organic rich deep-sea sediment (Webster et al., 2006a), shallow marine sediment (Oni et al., 2015), tidal flats (Webster et al., 2007) and methane hydrate bearing sediment (Reed et al., 2002; Inagaki et al., 2003, 2006). Their relative abundance often comprised higher than 50% of sequence libraries (Newberry et al., 2004; Inagaki et al., 2006; Carr et al., 2015). Those high abundances of *Atribacteria* in the methane rich sediments imply that this group was selected in high methane concentrations. In general, sediments of mud volcanoes contain high mathane concentrations and would be suitable enviromnent for *Atribacteira* as reported in Amsterdam mud volcano (Pachiadaki et al., 2011). The dominance of *Atribacteria* in MV#1 and MV#14 in this study was consistent with those previous studies.

At the Vodyanitskiy mud volcano in the Black Sea, it has been hypothesized that ANME-1 and ANME-2 archaea are associated with methane bubbles and participate in AOM in the anoxic water column (Schubert et al., 2006). In this study, we detected methane plumes together with atribacterial cells in the oxic water column, which is an environment very different from the deep sedimentary biosphere. We semi-quantitatively compared the frequency of 16S rRNA genes of OTU-1 between seawater and sediment samples, although the number of atribacterial cells in seawater visualized by CARD-FISH could not be directly compared to those in sediment samples, for which estimates were not technically possible because of the low *in situ* cell densities. Our results showed that, in methane plumes, the highest abundance of OTU-1 in the sequence library approached 1% relative abundance for MV#1 and MV#14 (**Figure 6**). These abundances are much higher than previously reported, where the highest relative abundance of sedimentary communities in the water column was 0.025% for normal sedimentary environments in the open ocean (Walsh et al., 2016).

The total abundance of 16S rRNA genes determined by microfluidic dPCR in methane plumes was 1.4 × 10^4^ copies/ml in samples from both SMVs. By multiplying the proportion of sequence by the gene abundance, we estimated the density of OTU-1 16S rRNA genes to be 1.2 × 10^2^ copies/ml and 1.1 × 10^2^ copies/ml for MV#1 and MV#14, respectively. The gene abundance in sediment pore water can be similarly estimated under the assumption that bulk density and porosity of the sediment are 1.8 g/ml and 50%, respectively (i.e., using shipboard results available in the Cruise Report of D/V *Chikyu* training cruise CK09-01, Leg. 1^2^). The estimated abundance of OTU-1 16S rRNA genes in the topmost sediment of MV#1 and MV#14 are 4.4 × 10^3^ and 6.3 × 10^3^ copies/ml pore water, respectively, which are only 37- and 57-fold higher than those in the methane plumes in the water column at 32 and 39 m above the respective SMVs. Given these estimates, we infer that there could be a supply of substantial amounts of the subseafloor microbial community to the overlying seawater, for which we propose the term “deep-biosphere plume.”

Once sedimentary microbial components are released into the water column, it is likely that they are transported via currents as “deep-biosphere seeds” into the ocean, perhaps over long distances, as previously discussed for thermospores found in arctic sediments (Hubert et al., 2009). Nevertheless, at this point, it is difficult to quantify the deep-biosphere seeds, constantly or episodically dispersed from SMVs along with methane and fluids. To expand our understanding of this interesting phenomenon involving geosystem–ecosystem interactions will require continuous monitoring of the geobiological system over a wide spatial range through future deep-sea research and ocean drilling opportunities.

In this study, by using the Atri578 probe targeting *Atribacteria* for CARD-FISH, we confirmed the presence of sedimentary *Atribacteria* in methane plumes (**Figure 7**). The detected atribacterial cells were individualized cocci (diameter approximately 1.0 μm), not forming aggregates or attached to any particulate matter. Our CARD-FISH observations suggest that the *Atribacteria* are generally free-living in the sediment pore water rather than tightly attached to the minerals. Because CARD-FISH detects rRNA inside individual cells, the detected cells may have been still alive and in their *in situ* metabolic state. This led us to hypothesize that deep-biosphere seeds of *Atribacteria* will colonize new habitats on the seafloor and revive to start growing, freshly deposited in a sedimentary habitat after traveling a long distance in the ocean.

Previous studies of the partial genome sequence of *Atribacteria* indicated that they are heterotrophic anaerobes that ferment organic acids, such as propionate, and produce the fermentation products acetate, ethanol, and CO_2_ (Carr et al., 2015; Nobu et al., 2016). Their fermentation products may therefore directly or syntrophically support methanogenesis or acetogenesis; that is, the terminal metabolic processes in the anoxic heterotrophic microbial ecosystem. In addition, it is worth noting that there have been no functional genes for methanotrophy so far identified in the known *Atribacteria*-derived genome sequences. This suggests that there may be little or no biogeochemical contributions from *Atribacteria* in methane plumes if they are just surviving and drifting in the water column. Their biogeochemical activities would resume when these deep-biosphere seeds are safely redeposited on the seafloor and buried back into the anoxic sedimentary habitat.

The number of cells in MV#1 and MV#14 sediment samples was much lower than in well-stratified sediment core samples from Pacific margin sites. In addition, the shallower mud volcano sediment in this study contained only 10^2^–10^5^ cells/g-wet, which is several orders of magnitude lower than the 10^8^–10^9^ cells/cm^3^ in very active and water-rich SMVs (e.g., Håkon Mosby mud volcano; Losekann et al., 2007). This implies that cell densities in the deeper zones of MV#1 and MV#14 are possibly much lower than in other, more active SMVs. Because the SMVs near Tanegashima originated from Eocene strata (Ujiie, 2000), it is possible that the low cell concentrations in the mud volcano sediment at this site can be explained by sediment matrices that are too deep and too old to support microbial life, and the geosystem may lack both available electron donors and acceptors. This, however, is still an open question.

## Conclusion

In this study, we investigated microbial communities at two SMVs off Tanegashima Island, Japan, using second generation sequencing, microfluidic digital PCR, image-based cell counts, and CARD-FISH techniques. Beta diversity of sediment and water-column microbial communities, as well as cell-count and gene quantification data, consistently indicated that microbial community compositions in mud volcano sediments are very distinct from those in well-stratified marginal sediments and water columns. Sedimentary *Atribacteria* were present in methane plumes in the overlying water column, suggesting microbial dispersal from the deep sedimentary biosphere to the overlying hydrosphere through submarine mud volcano activity. These findings suggest that a transmigration of “deep-biosphere seeds” may occur between the Earth’s surface and subsurface biospheres.

## Author Contributions

TH, TT, AI, YM, HM, JA, and FI sampled and pretreated a core sample. HM conducted surveys at the sampling sites. TH carried out molecular works as DNA extraction, sequencing and quantification of 16S rRNA gene, and data analysis. TT, AI, and KO performed measurement of methane and sulfate concentration. TH and YM carried out microscopic works including cell count and CARD-FISH. TH and FI designed of the work and drafted the manuscript, which was critically revised by all the other authors. The final manuscript was approved by all authors.

## Conflict of Interest Statement

The authors declare that the research was conducted in the absence of any commercial or financial relationships that could be construed as a potential conflict of interest.

## Footnotes

1 http://www.drive5.com/usearch/

2 www.godac.jamstec.go.jp
