# Supplementary material for: Atribacteria from the Subseafloor Sedimentary Biosphere Disperse to the Hydrosphere through Submarine Mud Volcanoes
Source: Front Microbiol. 2017 Jun 20;8:1135. doi: 10.3389/fmicb.2017.01135 (PMC5476839; doi:10.3389/fmicb.2017.01135)
Supplement: Supplementary file 1 [file Table_1.DOCX]

**Supplementary Table 1 Potential contaminated sequences removed in this study**

**Top 10 families appeared in the negative control**

Bacteria;Firmicutes;Bacilli;Bacillale;Lactobacillales;Streptococcaceae

Bacteria;Proteobacteria;Gammaproteobacteria;Enterobacteriales;Enterobacteriaceae

Bacteria;Proteobacteria;Gammaproteobacteria;Pseudomonadales;Pseudomonadaceae

Bacteria;Proteobacteria;Gammaproteobacteria;Pseudomonadales;Moraxellaceae

Bacteria;Fusobacteria;Fusobacteriia;Fusobacteriales;Fusobacteriaceae

Bacteria;Proteobacteria;Alphaproteobacteria;Rhizobiales;Methylobacteriaceae

Bacteria;Proteobacteria;Alphaproteobacteria;Sphingomonadales;MN_122.2a

Bacteria;Proteobacteria;Alphaproteobacteria;Sphingomonadales;Sphingomonadaceae

Bacteria;Proteobacteria;Betaproteobacteria;Burkholderiales;Comamonadaceae

Bacteria;Proteobacteria;Deltaproteobacteria;SAR324_clade(Marine_group_B);unclassified

**Human-core microbiome in Li et al., 2013**

Bacteria;Actinobacteria;Actinobacteria;Actinomycetales;Actinomycetaceae

Bacteria;Actinobacteria;Actinobacteria;Corynebacteriales;Corynebacteriaceae

Bacteria;Bacteroidetes;Flavobacteria;Flavobacteriales;Flavobacteriaceae

Bacteria;Firmicutes;Clostridia;Clostridiales;Lachnospiraceae

Bacteria;Firmicutes;Bacilli;Lactobacillales;Lactobacillaceae

Bacteria;Proteobacteria;Betaproteobacteria;Neisseriales;Neisseriaceae

Bacteria;Proteobacteria;Gammaproteobacteria;Pasteurellales;Pasteurellaceae

Bacteria;Bacteroidetes;Bacteroidia;Bacteroidales;Porphyromonadaceae

Bacteria;Bacteroidetes;Bacteroidia;Bacteroidales;Prevotellaceae

Bacteria;Actinobacteria;Actinobacteria;Propionibacteriales;Propionibacteriaceae

Bacteria;Bacteroidetes;Bacteroidia;Bacteroidales;Rikenellaceae

Bacteria;Firmicutes;Bacilli;Bacillales;Staphylococcaceae

Bacteria;Firmicutes;Negativicutes;Selenomonadales;Veillonellaceae

[1] Li, K., Bihan, M., Methe, B.A., Analyses of the stability and core taxonomic memberships of the human microbiome, PLoS One, 8 (2013) e63139.
