# Supplementary material for: Atribacteria from the Subseafloor Sedimentary Biosphere Disperse to the Hydrosphere through Submarine Mud Volcanoes
Source: Front Microbiol. 2017 Jun 20;8:1135. doi: 10.3389/fmicb.2017.01135 (PMC5476839; doi:10.3389/fmicb.2017.01135)
Supplement: Supplementary file 2 [file Table_2.DOCX]

Supplementary Table 2 Proportion of the removal sequences as potential contaminants

|  | Number of obtained sequences | Number of sequences after removing potential contaminants | Remaining sequences (%) |
| --- | --- | --- | --- |
| VTF2_3_1 | 53648 | 43559 | 81.2 |
| VTF2_3_2 | 41965 | 34425 | 82.0 |
| VTF2_4_1 | 52847 | 16275 | 30.8 |
| VTF2_4_2 | 13183 | 2037 | 15.5 |
| VTF2_4_3 | 63151 | 15006 | 23.8 |
| VTF2_4_4 | 55132 | 37258 | 67.6 |
| VTF2_5_2 | 69427 | 21333 | 30.7 |
| VTF2_5_3 | 70664 | 21955 | 31.1 |
| VTF2_5_4 | 49890 | 12882 | 25.8 |
| VTF2_6_1 | 22207 | 6525 | 29.4 |
| VTF2_6_2 | 104468 | 46882 | 44.9 |
| VTF2_6_4 | 10102 | 3186 | 31.5 |
| VTF2_W_1 | 81974 | 77571 | 94.6 |
| VTF2_W_2 | 47018 | 44459 | 94.6 |
| VTF2_W_3 | 36648 | 34424 | 93.9 |
| VTF2_W_4 | 36213 | 33755 | 93.2 |
| VTF2_W_5 | 117416 | 110182 | 93.8 |
| VTF2_W_6 | 88589 | 83844 | 94.6 |
| VTF2_W_7 | 57080 | 53593 | 93.9 |
| VTF2_W_8 | 55464 | 52576 | 94.8 |
| VTF5_4_1 | 29553 | 27765 | 93.9 |
| VTF5_4_2 | 50611 | 47121 | 93.1 |
| VTF5_4_3 | 19363 | 17788 | 91.9 |
| VTF5_4_4 | 117311 | 51324 | 43.8 |
| VTF5_5_1 | 89469 | 71744 | 80.2 |
| VTF5_5_2 | 110911 | 87601 | 79.0 |
| VTF5_5_3 | 39087 | 20124 | 51.5 |
| VTF5_5_4 | 100812 | 19449 | 19.3 |
| VTF5_6_1 | 33305 | 12830 | 38.5 |
| VTF5_6_2 | 38044 | 28081 | 73.8 |
| VTF5_6_4 | 20907 | 13247 | 63.4 |
| VTF5_W_1 | 74291 | 70153 | 94.4 |
| VTF5_W_2 | 66144 | 62397 | 94.3 |
| VTF5_W_3 | 41090 | 38635 | 94.0 |
| VTF5_W_4 | 45561 | 41915 | 92.0 |
| VTF5_W_5 | 70828 | 66466 | 93.8 |
| VTF5_W_6 | 38720 | 36301 | 93.8 |
| VTF5_W_7 | 14614 | 13251 | 90.7 |
| VTF5_W_8 | 129746 | 119824 | 92.4 |
