# Supplementary material for: Atribacteria from the Subseafloor Sedimentary Biosphere Disperse to the Hydrosphere through Submarine Mud Volcanoes
Source: Front Microbiol. 2017 Jun 20;8:1135. doi: 10.3389/fmicb.2017.01135 (PMC5476839; doi:10.3389/fmicb.2017.01135)
Supplement: Supplementary file 3 [file Image_1.PDF]

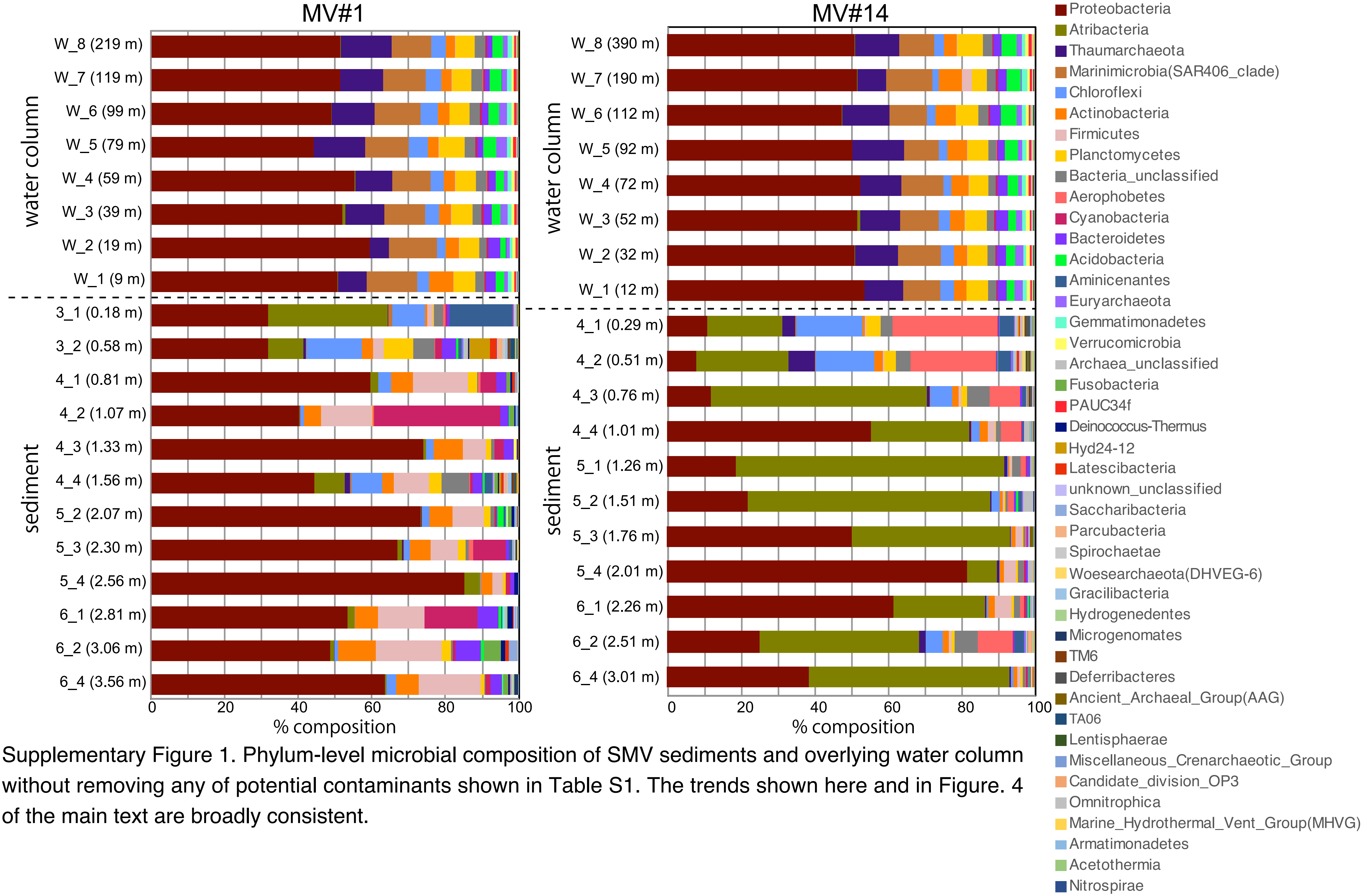

Supplementary Figure 1. Phylum-level microbial composition of SMV sediments and overlying water column without removing any of potential contaminants shown in Table S1. The trends shown here and in Figure. 4 of the main text are broadly consistent.
