# Supplementary material for: Atribacteria from the Subseafloor Sedimentary Biosphere Disperse to the Hydrosphere through Submarine Mud Volcanoes
Source: Front Microbiol. 2017 Jun 20;8:1135. doi: 10.3389/fmicb.2017.01135 (PMC5476839; doi:10.3389/fmicb.2017.01135)
Supplement: Supplementary file 4 [file Image_2.PDF]

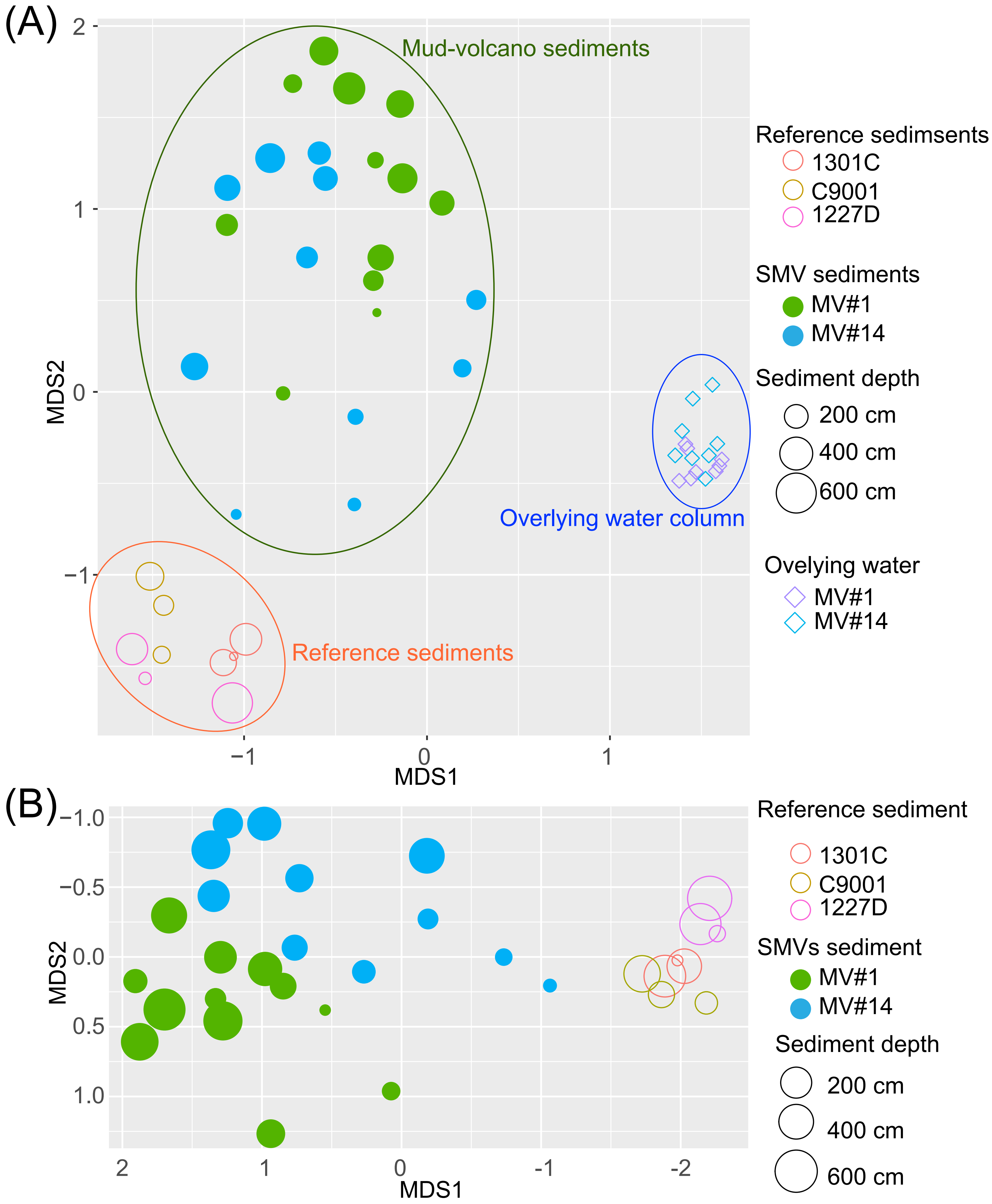

Supplementary Figure 2. Non-metric multidimensional scaling (NMDS) plot of microbial communities from submarine mud volcano (SMV) sediment (circles) and the overlying water column (diamonds), with stratified marine sediment communities included for reference, based on operational taxonomic unit (OTU) composition . Any of potential contaminants (see. Table S1) were not removed from sequence libraries. The size of the circles for sediment samples, including SMV sediment is proportional to sediment depth. (A) NMDS plot of all the samples including the water column (B) Only SMV and reference sediments.
