# Supplementary material for: Atribacteria from the Subseafloor Sedimentary Biosphere Disperse to the Hydrosphere through Submarine Mud Volcanoes
Source: Front Microbiol. 2017 Jun 20;8:1135. doi: 10.3389/fmicb.2017.01135 (PMC5476839; doi:10.3389/fmicb.2017.01135)
Supplement: Supplementary file 5 [file Image_3.PDF]

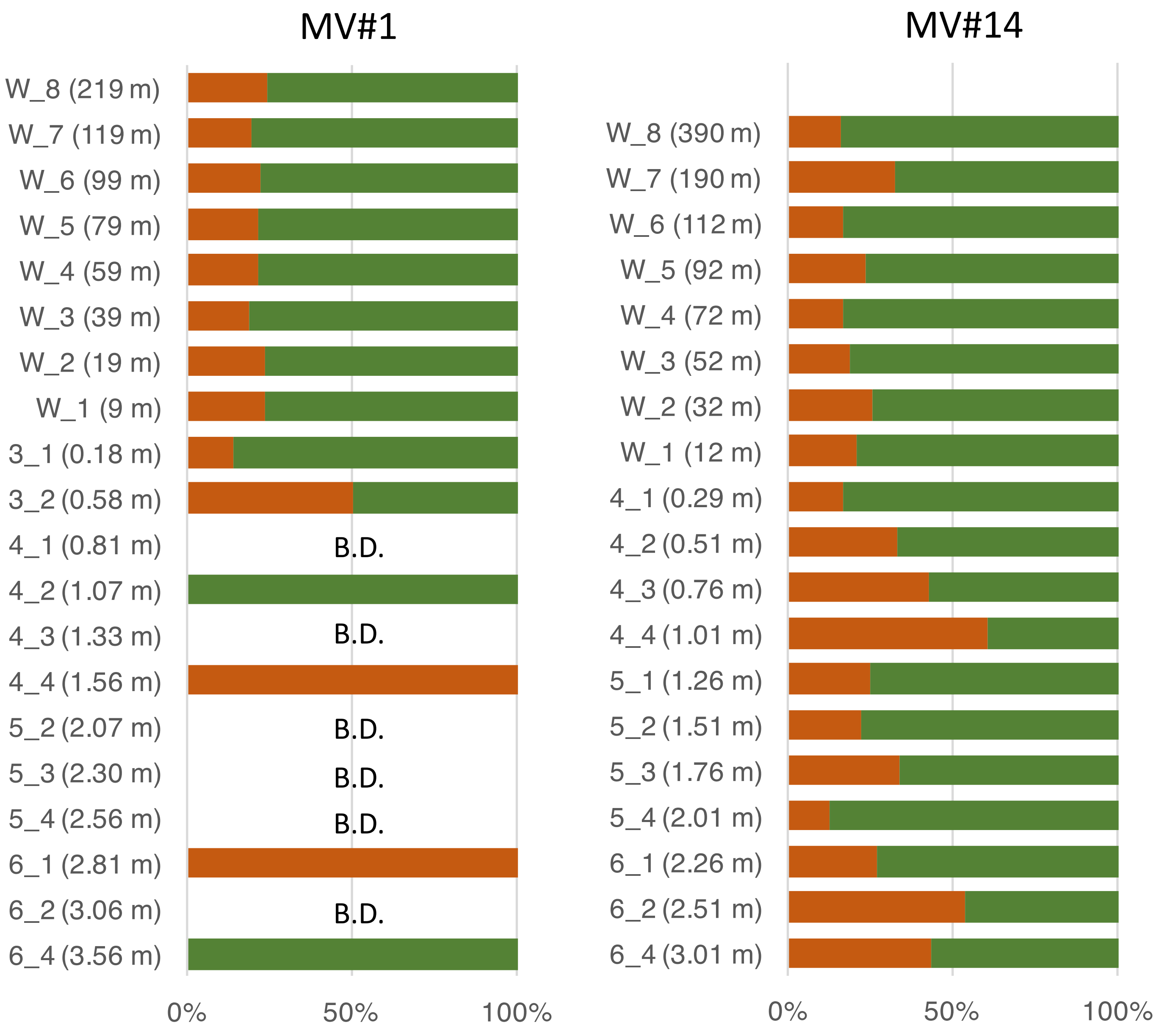

Supplementary Figure 3. Proportion of bacterial (shown in brown) and archaeal (green) 16S rRNA gene quantified by dPCR shown in Fig. 3. B.D. below detection limit.
