# Supplementary material for: Atribacteria from the Subseafloor Sedimentary Biosphere Disperse to the Hydrosphere through Submarine Mud Volcanoes
Source: Front Microbiol. 2017 Jun 20;8:1135. doi: 10.3389/fmicb.2017.01135 (PMC5476839; doi:10.3389/fmicb.2017.01135)
Supplement: Supplementary file 7 [file Image_5.PDF]

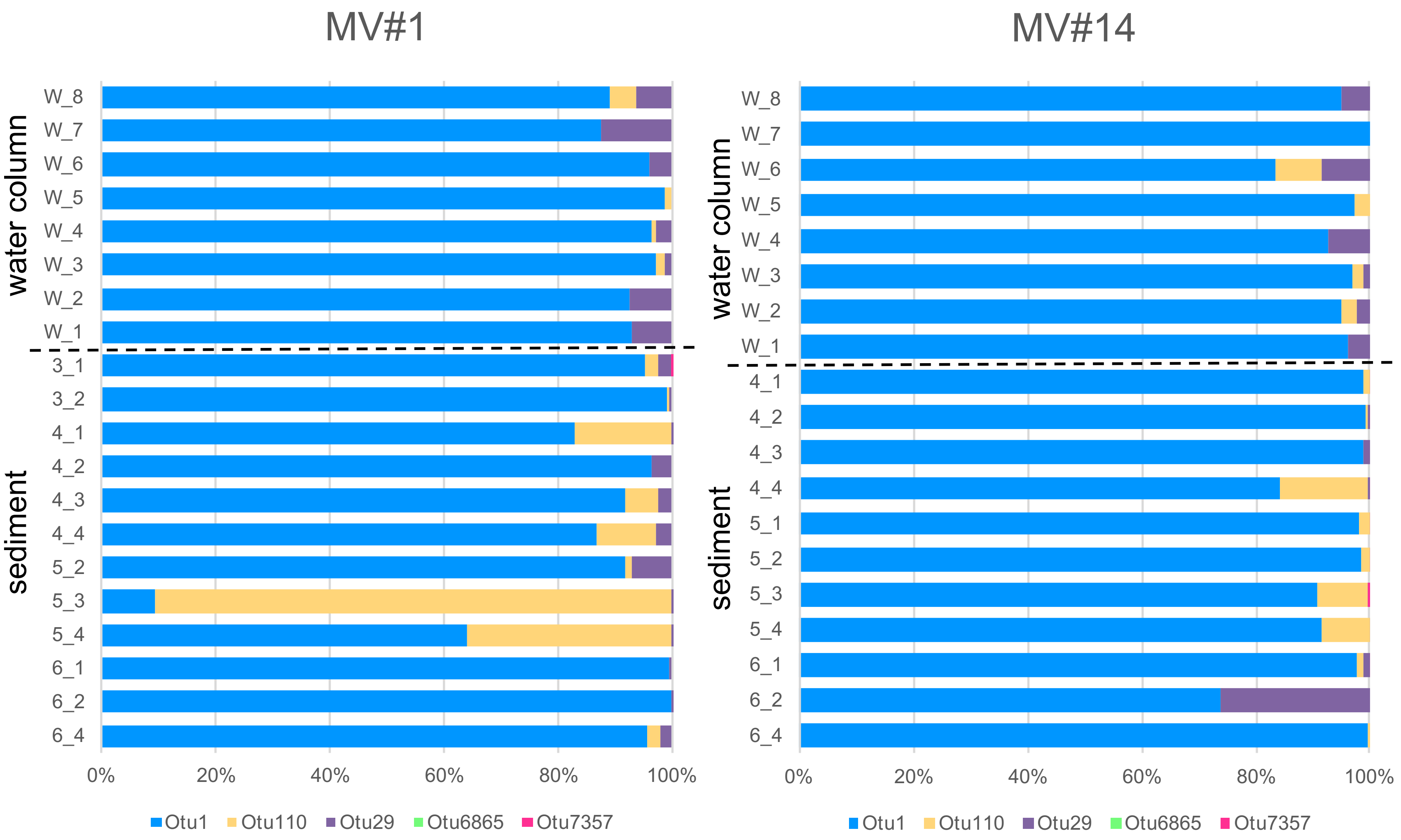

Supplementary Figure 5. Composition of OTUs affiliated with *Atribacteria* in each sample. In total, 4 OTUs were affiliated with *Atribacteria* in this study.
