# Supplementary material for: Atribacteria from the Subseafloor Sedimentary Biosphere Disperse to the Hydrosphere through Submarine Mud Volcanoes
Source: Front Microbiol. 2017 Jun 20;8:1135. doi: 10.3389/fmicb.2017.01135 (PMC5476839; doi:10.3389/fmicb.2017.01135)
Supplement: Supplementary file 8 [file Image_6.PDF]

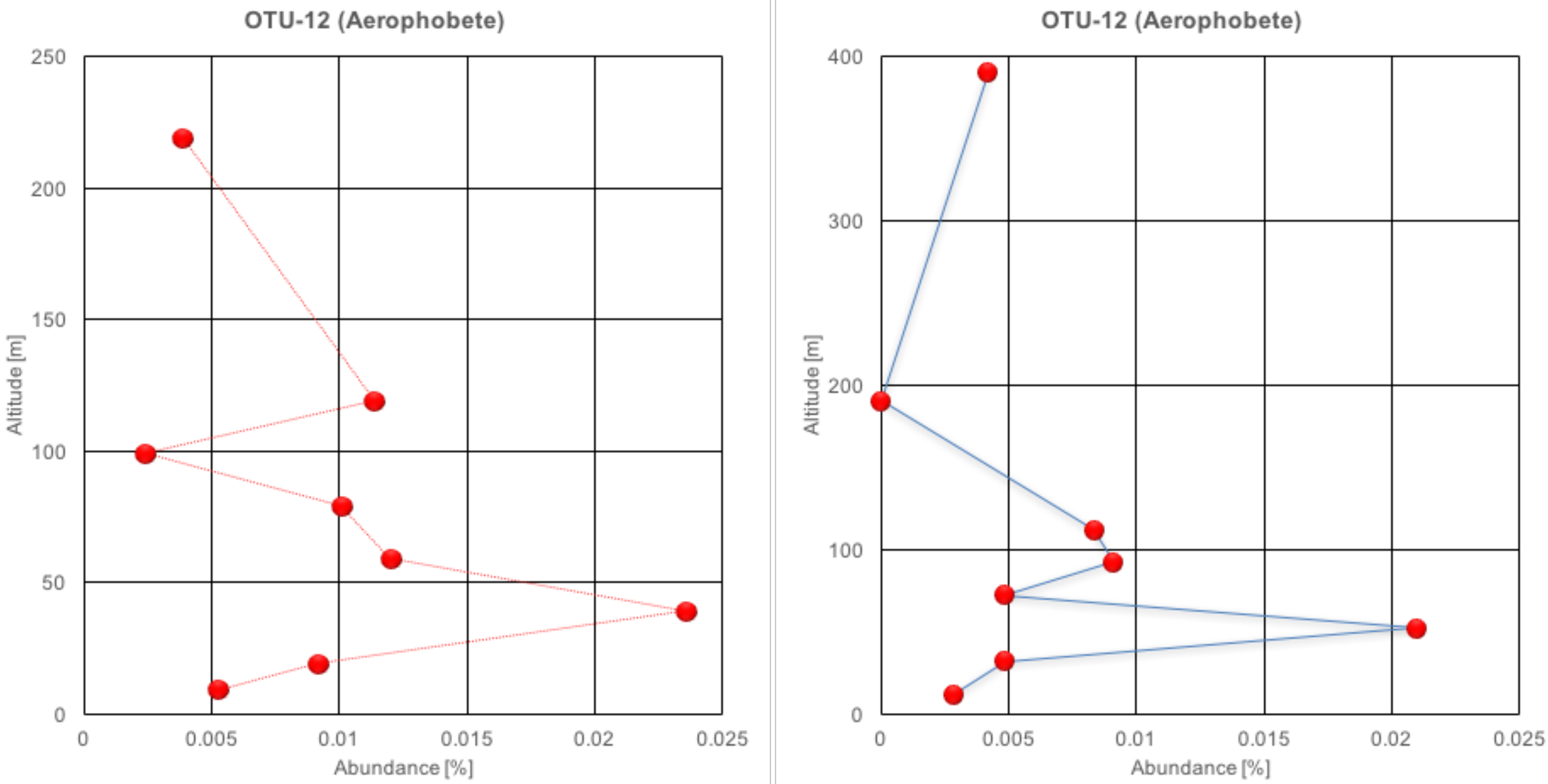

Supplementary Figure 6. Depth profile of the abundance of Otu12 in the water column. Otu 12 is the second most abundant OTU in the sediment samples.
